# Macrostemonoside I alleviates metabolic dysfunction-associated steatotic liver disease in mice: potential involvement of SCAP/SREBP2 signaling in cholesterol metabolism

**DOI:** 10.3389/fmed.2026.1983132

**Published:** 2026-10-14

**Authors:** Jiyuan Su, Shouyu Lin, Chuanshen Jiang, Huimin Lin, Yunhua Yu, Wen Wang

**Affiliations:** 1 Fuzong Clinical Medical College of Fujian Medical University, Fuzhou, China; 2 Department of Geriatrics, 900th Hospital of PLA Joint Logistic Support Force, Fuzhou, China; 3 Outpatient Department, 900th Hospital of PLA Joint Logistic Support Force, Fuzhou, China; 4 Department of Gastroenterology, 900th Hospital of PLA Joint Logistic Support Force, Fuzhou, China

**Keywords:** cholesterol metabolism, macrostemonoside I, metabolic dysfunction-associated steatotic liver disease, SREBP cleavage-activating protein, sterol regulatory element-binding protein 2

## Abstract

**Introduction:**

The progression of metabolic dysfunction-associated steatotic liver disease (MASLD) involves lipotoxicity and inflammation from disordered cholesterol metabolism. Macrostemonoside I (MI), an *Allium* saponin monomer, may regulate cholesterol metabolism, but its role in MASLD is unclear.

**Methods:**

MASLD was induced in mice by a 6-week choline-deficient high-fat diet (CDAHFD) (1-week gradual transition to CDAHFD and 5 weeks of continuous CDAHFD feeding), followed by treatment with MI (2, 4, 8 mg/kg/d), atorvastatin, or vehicle for 30 days. Body weight, liver index, serum levels of HDL-C, ALT, AST and inflammatory cytokines were measured. Hepatic cholesterol content, histopathology (H&E, Oil Red O, Sirius red, Immunofluorescent staining), oxidative stress markers, transcriptome and the expression of genes/proteins related to cholesterol metabolism and fibrosis were analyzed.

**Results:**

MI reduced liver index, ameliorated hepatic ballooning and steatosis, and decreased hepatic total cholesterol and free cholesterol, as well as serum ALT/AST levels, but had no significant effect on HDL-C. It reduced hepatic CD11b⁺ infiltration and serum IL-1β, IL-6, TNF-α levels. Liver fibrosis area, and the fibrosis markers COL1A1 and α-SMA, were downregulated. MI also decreased hepatic ROS and MDA, and increased SOD activity. Furthermore, MI treatment was associated with changes in the cholesterol transcriptome and with significant reductions in both the mRNA and protein levels of the cholesterol metabolism-related molecules SCAP and SREBP2.

**Discussion:**

MI might alleviate MASLD by reducing cholesterol accumulation, suppressing inflammation, ameliorating fibrosis, and mitigating oxidative stress, potentially via SCAP/SREBP2 pathway. However, causality remains to be determined.

## 1 Introduction

Metabolic dysfunction-associated steatotic liver disease (MASLD), formerly termed nonalcoholic fatty liver disease (NAFLD), has emerged as the most prevalent chronic liver disorder worldwide, affecting approximately 25% of the global population (1). This surge is closely linked to the rising epidemics of obesity and metabolic syndrome (MetS), for which MASLD is considered the hepatic manifestation. Importantly, MASLD not only progresses from simple steatosis to steatohepatitis, fibrosis, cirrhosis, and hepatocellular carcinoma (2, 3), but also increases the risk of extrahepatic complications such as type 2 diabetes, cardiovascular disease, and chronic kidney disease. To date, no pharmacological agent has been approved for MASLD by either the U.S. Food and Drug Administration or the European Medicines Agency, representing a major unmet clinical need. In 2020, an international expert consensus redefined MASLD based on the presence of hepatic steatosis along with overweight/obesity, type 2 diabetes, or metabolic dysregulation, underscoring that metabolic dysfunction is a non-negligible driver of the disease (4). Consequently, lipid-lowering and glucose-controlling medications such as statins, pioglitazone, and metformin are considered therapeutic options for MASLD, despite the lack of dedicated indications (5).

Among the pathological drivers of MASLD, lipotoxicity-induced liver injury plays a critical role. As major lipotoxic molecules, the accumulation of cholesterol and its metabolites within hepatic tissues accelerates MASLD progression. While cholesterol is essential for membrane integrity and the synthesis of bile acids, vitamin D, and steroid hormones, its homeostasis is crucial for normal cellular function (6, 7). Hypercholesterolemia promotes simple steatosis, accelerates the transition to steatohepatitis, and may even contribute to MASLD-related hepatocellular carcinoma. Notably, a high-cholesterol diet can activate macrophages and create a pro-inflammatory hepatic microenvironment (8).

In traditional Chinese medicine (TCM) theory, MASLD is primarily attributed to “turbid stasis” and “static blood”, with underlying pathogenesis involving spleen dysfunction, liver qi stagnation, and phlegm-stasis interaction (9). The classic Chinese formula Gualou Xiebai Decoction (GXD), known for its principle of “synchronically treating phlegm and blood stasis,” has been shown to possess lipid-lowering, anti-atherosclerotic, antioxidant, anti-apoptotic, and anti-inflammatory properties (10, 11). Our preliminary experiments demonstrated that GXD significantly reduced plasma and hepatic cholesterol levels in atherosclerotic model mice (12). As the principal active components of *Allium* macrostemon Bunge (one of the key herbs in GXD), *Allium* saponins exhibit antiplatelet, antioxidant, lipid-lowering, and antibacterial activities (13). Xie et al. reported that *Allium* saponins ameliorate fasting blood glucose, hepatic glycogen, serum total cholesterol, and visceral fat accumulation in mice fed with a high-fat diet (14).

Despite these promising findings, the role of 26-*O*-*β*-D-glucopyranosyl-22-hydroxy-5*β*-furost-25(27)-ene-12-one-3*β*, 26-diol 3-*O*-*β*-D-glucopyranosyl(1 → 2)-*β*-D-galactopyranoside [macrostemonoside I, MI, a monomeric compound derived from *Allium* saponins (15), Figure 1].

**Figure 1:**
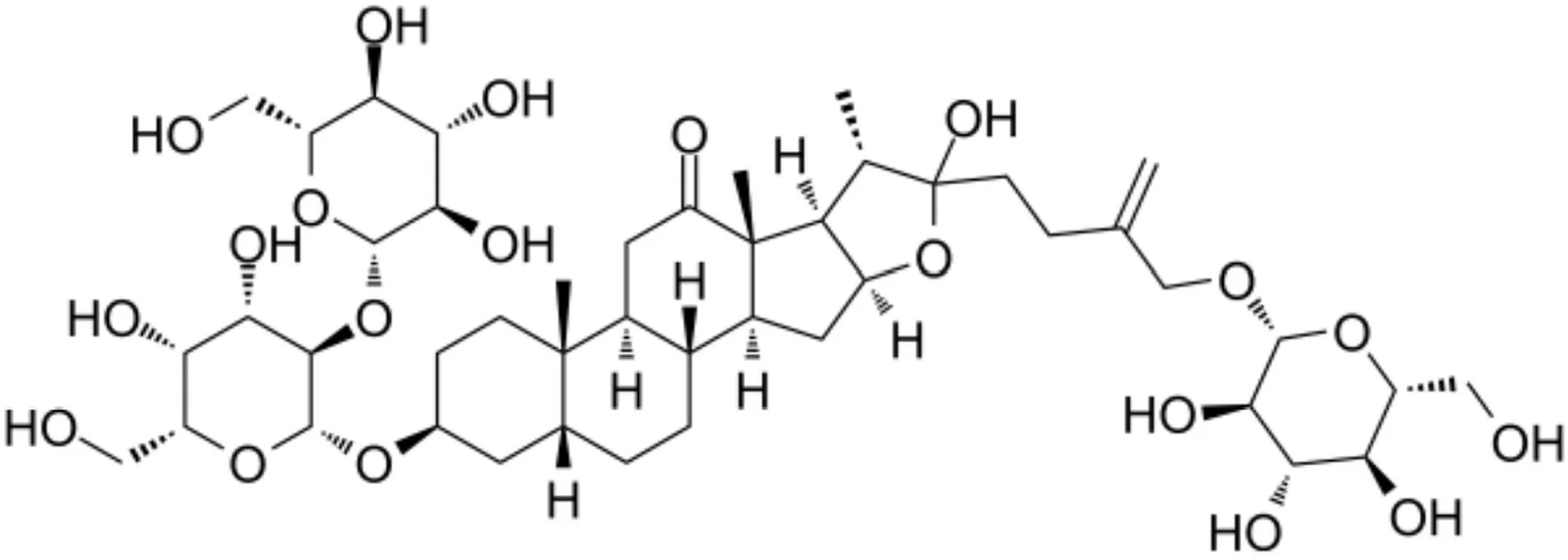
Structure of macrostemonoside I.

in MASLD remains unknown. Given the critical role of hepatic cholesterol accumulation in MASLD pathogenesis and the cholesterol-lowering effect of GXD, we hypothesize that MI alleviates MASLD by regulating hepatic cholesterol metabolism. In the present study, we established animal model of MASLD to investigate the effects of MI and to elucidate the underlying molecular mechanisms. These findings provide a preclinical rationale for further investigation of MI, an active constituent derived from TCM, as a potential therapeutic candidate for this high-risk population.

## 2 Materials and methods

### 2.1 Reagents

Supplementary Text S1 in the Supporting Information provides specific information regarding the reagents used in this study.

### 2.2 Mouse MASLD model and treatment

Male C57BL/6J mice at six weeks of age were obtained from Biotechnology Co., Ltd. (Beijing, China). For the experiment, these mice were randomly allocated into six groups (*n* = 5 per group): control, MASLD, MASLD plus atorvastatin (MASLD + ATS), and MASLD plus low-, medium-, or high-dose MI (2 mg/kg/day, 4 mg/kg/day, and 8 mg/kg/day, respectively; designated as MASLD + L-MI, MASLD + M-MI, and MASLD + H-MI). Before administration, MI powder was dissolved in sterile normal saline and administered by oral gavage in a volume of 0.5 mL per mouse once daily. The MI doses were selected based on a previously published study in which macrostemonoside A at 4 mg/kg/day for 30 days mildly to moderately inhibited elevated fasting blood glucose, liver glycogen, serum total cholesterol, and visceral fat accumulation in high-fat diet-fed C57BL/6 mice (14). Given the limited dose-response data available for MI, we adopted this reported effective dose as the medium-dose group, and set the low- and high-dose groups at half and twice this dose, respectively, to evaluate potential dose-dependent effects. The mice were housed under standard SPF conditions. The environment was regulated to maintain a temperature of 22 ± 2 ℃, a relative humidity of 50 ± 10%, and a standard 12-hour light/dark photoperiod. Mice were fed either a standard normal chow diet (5% fat, 20% protein, 60% carbohydrates, 5% fibre, 10% vitamin) or a choline-deficient, L-amino acid-defined high-fat diet (CDAHFD, A06071302; Xietong Bio-engineering Co., Ltd., China) containing 37% fat, 32% carbohydrate, 23% protein by weight (w/w), and total 5.20 kcal/g (as provided by the manufacturer). Following a 1-week dietary acclimatization period with a gradual transition to the CDAHFD, the experimental feeding was continued for 5 weeks (total MASLD induction period: 6 weeks). After the MASLD model was established, the respective treatment groups received daily interventions via oral gavage for 30 days: the MASLD + ATS group was given atorvastatin calcium tablets (ground and suspended in normal saline) at a dose of 2.6 mg/kg/day, while the MASLD + L-MI, MASLD + M-MI, and MASLD + H-MI groups received low, medium, and high doses of MI, respectively. Body weight was recorded weekly throughout the experiment. Following this, at the conclusion of the treatment, all animals underwent a 12-hour fasting period (water permitted) prior to being humanely euthanized with isoflurane. Following collection, blood samples were centrifuged (1,000 g, 10 min, 4 °C) to isolate serum, which was then stored for subsequent biochemical assays. Fresh tissue specimens were snap-frozen in liquid nitrogen and maintained at −80 °C for future studies. The overall experimental timeline is depicted in Figure 2A. This study was performed in compliance with the ARRIVE guidelines and received ethical approval from the Institutional Animal Care and Use Committee of Fuzong Clinical Medical College of Fujian Medical University (Approval No. 2024-016).

**Figure 2:**
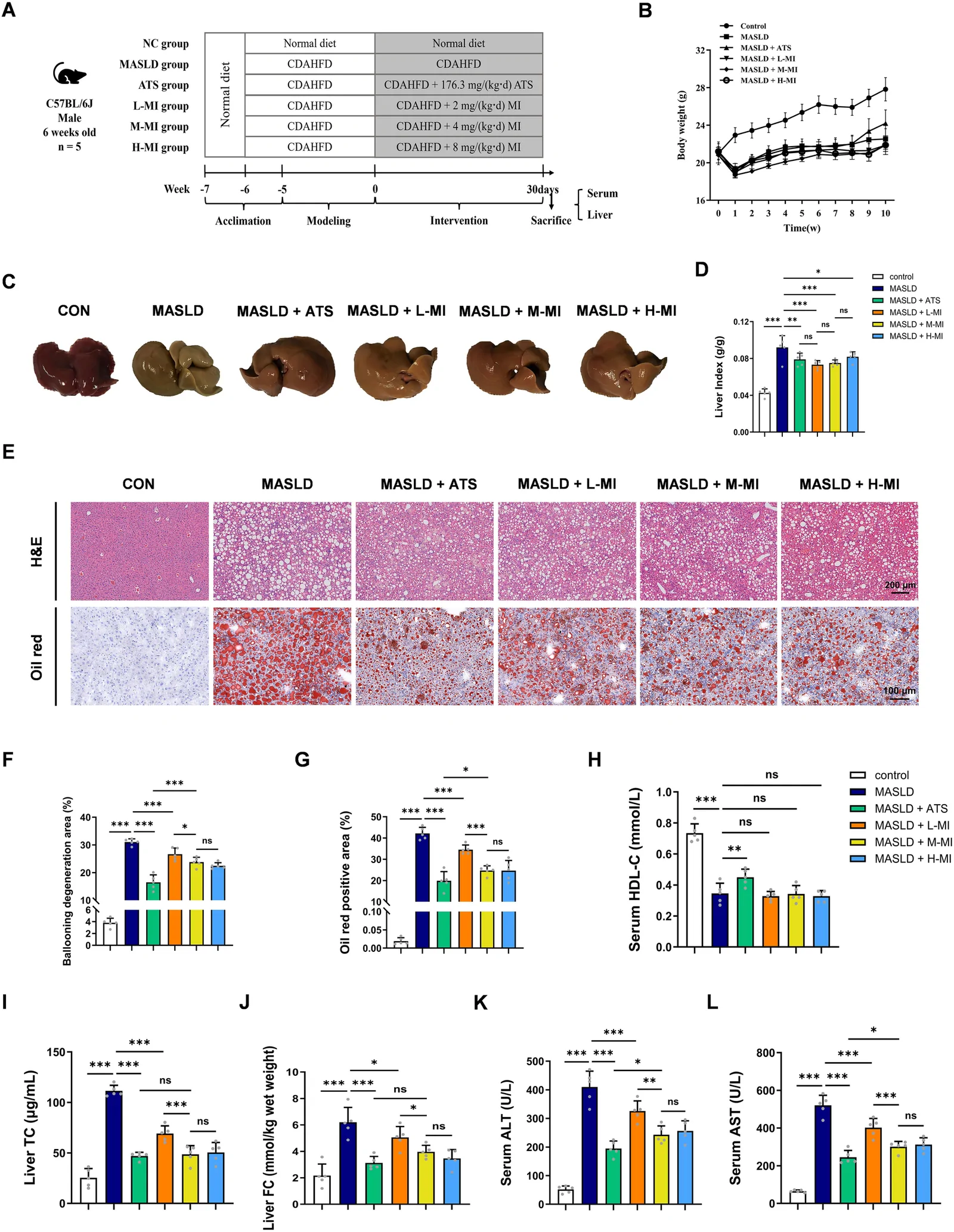
MI treatment attenuated hepatic cholesterol accumulation and improved liver function in MASLD mice (*n* = 5 biological replicates per group). **(A)** Experimental design of animal studies; **(B–D)** Body weight, gross morphology of representative mouse livers and liver index; **(E–G)** Representative images of H&E and Oil Red O staining and respective statistical analysis; **(H)** serum HDL-C level; **(I,J)** Contents of hepatic TC and FC; **(K,L)** Levels of serum ALT and AST. Data are presented as mean ± SD. Statistical significance was determined by one-way ANOVA with Bonferroni's comparison test. Horizontal lines represent the statistical comparisons between the two connected groups. ns, not significant, *P < 0.05, **P < 0.01, ***P < 0.001.

### 2.3 Measurement of hepatic total and free cholesterol (TC/FC) content

Liver tissues were washed with ice-cold PBS to thoroughly remove residual blood, blotted dry, and weighed prior to homogenization. The tissues were minced into fine pieces and divided into two portions. One portion was homogenized in freshly prepared lysis buffer (1:30, w/v) using a glass homogenizer on ice for total cholesterol assay, and the other portion was homogenized in absolute ethanol for free cholesterol measurement. Subsequently, the homogenate was subjected to sonication using an ultrasonic cell disrupter until a clear lysate was obtained. The lysates were centrifuged at 10,000 × g for 5 min to collect the supernatant. Hepatic total and free cholesterol concentrations in the supernatant were determined using commercial assay kits according to the manufacturers' protocols. Absorbance was recorded at 450 nm for TC and 510 nm for FC. For TC, standard solutions at different concentrations were prepared, and a standard curve was generated based on their optical density (OD) values. The OD values of the samples were substituted into the standard curve to obtain their concentrations, expressed as μg/mL. For FC, the concentration was calculated as: FC = (ΔA1/ΔA2) × c × f/(m/V). ΔA1 is the sample OD minus the blank OD, ΔA2 is the standard OD minus the blank OD, c is the standard concentration (5.17 mmol/L), f is the dilution factor of the sample before being added to the assay system, m is the liver tissue weight (g), and V is the volume of anhydrous ethanol (mL). FC are expressed as mmol/kg wet weight.

### 2.4 Measurement of serum levels of high-density lipoprotein cholesterol (HDL-C), alanine aminotransferase (ALT) and aspartate aminotransferase (AST)

Blood was collected from mice by submandibular vein puncture. The samples were allowed to clot and subsequently centrifuged to obtain serum. Serum HDL-C, ALT and AST levels were quantified using an automated biochemical analyzer.

### 2.5 Measurement of serum inflammatory cytokine levels

Blood samples were obtained through puncture of the submandibular vein and allowed to clot at room temperature. Following clotting, serum was separated by centrifugation. Levels of the proinflammatory cytokines IL-1β, IL-6, and TNF-α in serum were quantified using commercially available enzyme-linked immunosorbent assay (ELISA) kits, following the protocols provided by the manufacturers. Absorbance measurements were taken at a wavelength of 450 nm.

### 2.6 Tissue characterization and biochemical assays

Liver tissues were analyzed to assess histopathological, biochemical, and molecular changes. Techniques included haematoxylin and eosin (H&E) and Oil Red O staining for pathological changes, Sirius red staining for hepatic fibrosis, ventricular oxidative stress assessment via dihydroethidium staining and measuring malondialdehyde (MDA) and superoxide dismutase (SOD) levels, immunohistochemical staining for hepatic fibrosis (α-SMA, COL1A1), immunofluorescent staining for CD11b^+^ cells, transcriptome sequencing, qRT-PCR and Western blotting for gene expression (AR, SCAP, SREBP2, β-Actin). Detailed protocols for all assays are provided in the SI (Supplementary Text S2).

### 2.7 Statistical analysis

Data normality was confirmed by the Shapiro-Wilk test and histogram assessment. The data met the homogeneity of variance, which was assessed using Levene's test, and are presented as mean ± standard deviation. Statistical analyses were performed using SPSS (version 21.0) and GraphPad Prism (version 9.0). The difference among groups were compared using one-way analysis of variance (ANOVA) with Bonferroni's comparison test. Multiple-comparison correction was consistently applied to all *post hoc* pairwise comparisons. Statistical significance was set at *p* < 0.05.

## 3 Results

### 3.1 Mi treatment attenuated hepatic cholesterol accumulation and improved liver function

During the experimental period, the body weight of mice in all groups showed a general upward trend (Figure 2B). Gross examination of mouse livers revealed that the control group exhibited a dark red color, whereas the MASLD group showed a yellowish-brown appearance with a glossier surface, enlarged size, and blunted edges. The livers of the ATS and MI intervention groups demonstrated varying degrees of improvement (Figure 2C). At the end of the study, the liver index was calculated based on liver weight and compared among groups. Compared with the control group, mice in the MASLD group showed a significant increase in liver index, which was notably reduced by both ATS and various doses of MI, with no significant differences observed among the ATS and MI treatment groups (Figure 2D). H&E staining revealed a marked increase in hepatocellular ballooning in MASLD mice compared to controls (*p* < 0.001). Both ATS and MI treatments significantly alleviated this morphological alteration. Moderate-dose MI produced a more pronounced improvement than low-dose MI (*p* = 0.017), while its effect was comparable to that of high-dose MI (*p* = 0.236). In this model, ATS treatment resulted in a greater reduction in ballooning area than moderate-dose MI. Consistent with the H&E findings, Oil Red O staining indicated a significant increase in hepatic lipid droplet area in the MASLD group (*p* < 0.001), which was effectively attenuated by ATS and all MI doses. Moderate-dose MI had a greater cholesterol ester- and triglyceride-lowering effect than low-dose MI (*p* < 0.001), but similar to high-dose MI (*p* = 0.985). Additionally, ATS treatment led to a greater reduction in these lipids than moderate-dose MI in this model (Figures 2E–G). In the MASLD group, serum HDL-C levels were markedly reduced, consistent with the MASLD diagnostic criteria, in which reduced HDL-C is one of the five cardiometabolic risk factors (16). ATS could ameliorate this reduction to a certain degree, whereas MI did not exert a significant impact on HDL-C in the MASLD model (Figure 2H). Hepatic TC and FC levels were significantly elevated in MASLD mice (TC: *p* < 0.001; FC: *p* < 0.001). Both ATS and MI significantly reduced both TC and FC, with moderate-dose MI being more effective than low-dose MI (TC: *p* < 0.001; FC: *p* = 0.037) and comparable to high-dose MI and ATS (Figures 2I,J). Serum ALT and AST levels were significantly higher in the MASLD group and were substantially lowered by ATS and all MI doses. Moderate-dose MI outperformed low-dose MI but showed similar efficacy to high-dose MI, whereas ATS showed a more pronounced effect than moderate-dose MI under the present conditions (Figures 2K,L). In summary, MI treatment effectively reduced intrahepatic cholesterol content and improved liver function in MASLD mice. Although some effects of MI were less pronounced than those of ATS in this model, MI and ATS may act through different mechanisms. Therefore, this comparison does not imply clinical superiority or inferiority. Among the MI doses tested, the moderate dose showed better activity than the low dose while the high dose was not significantly superior to the moderate dose. Therefore, the moderate dose of MI was selected for subsequent experiments.

### 3.2 MI treatment ameliorated hepatic inflammation

The activation and differentiation of myeloid cells in inflammatory settings upregulate CD11b expression, making it a useful marker for tracking disease progression and therapeutic efficacy. Immunofluorescence staining revealed that the relative fluorescence intensity of CD11b in the liver tissue of MASLD mice was significantly higher than that in the control group. Both ATS and the optimal dose of MI significantly reduced this intensity, with no statistically significant difference observed between the two treatments (Figures 3A,B). Serum inflammatory factor analysis showed that levels of IL-1β, IL-6, and TNF-α were markedly elevated in MASLD mice. These increases were ameliorated by both ATS and MI treatment. ATS and MI showed comparable effects in reducing IL-1β and TNF-α levels in this model, while ATS led to a greater reduction in IL-6 than MI under the present experimental conditions (Figures 3C–E). In summary, the optimal dose of MI alleviated hepatic inflammation and reduced the production of inflammatory cytokines in MASLD mice. Although both ATS and MI demonstrated anti-inflammatory effects in this model, their effects differed for certain cytokines.

**Figure 3:**
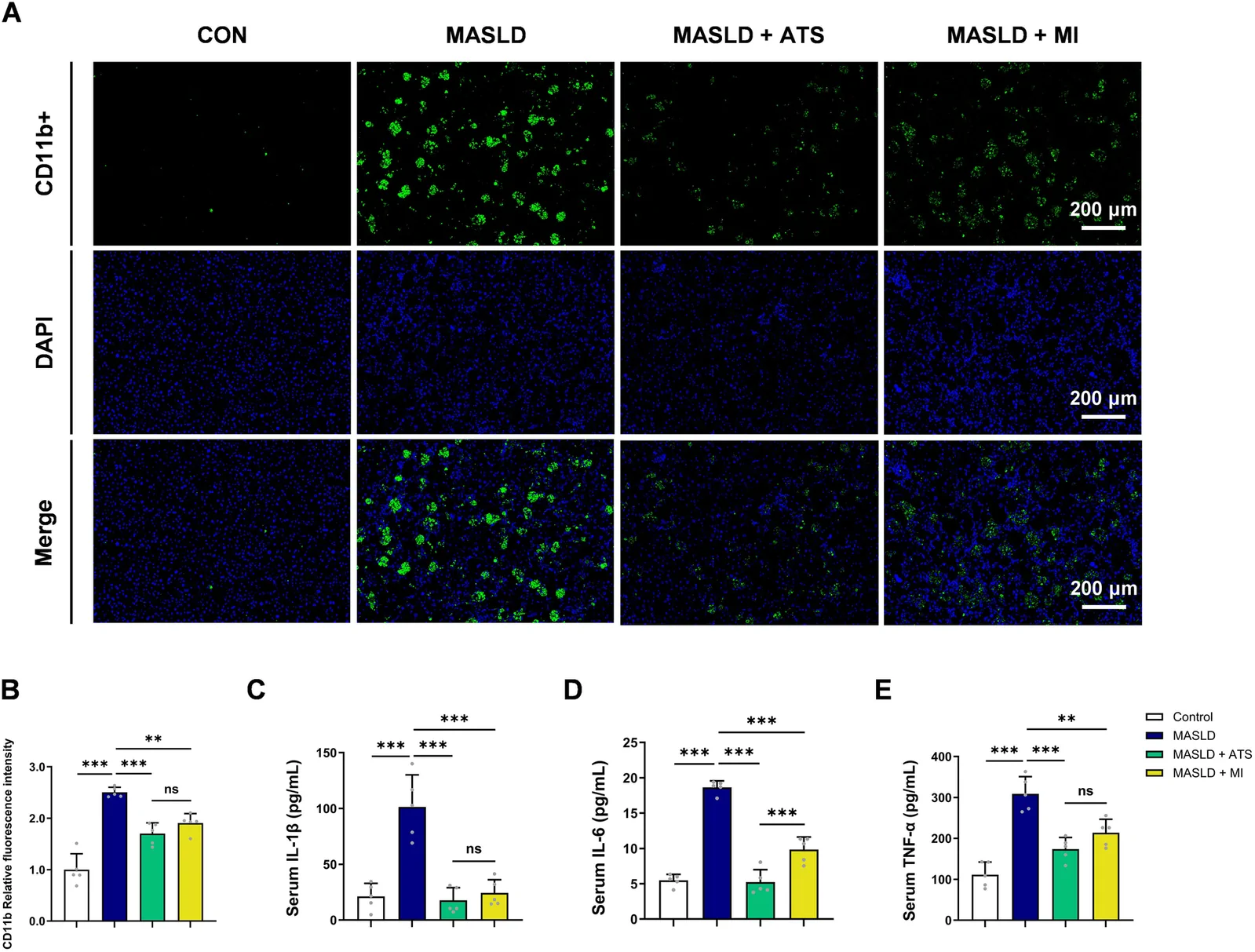
MI treatment (4 mg/kg/day) ameliorated hepatic inflammation in mice with MASLD (*n* = 5 biological replicates per group). **(A,B)** Representative images of immunofluorescence staining and statistical analysis of relative fluorescence intensity of CD11b; **(C–E)** Levels of serum IL-1β, IL-6, and TNF-α. Data are presented as mean ± SD. Statistical significance was determined by one-way ANOVA with Bonferroni's comparison test. Horizontal lines represent the statistical comparisons between the two connected groups. ns, not significant, **P < 0.01, ***P < 0.001.

### 3.3 MI treatment attenuated hepatic fibrosis

Based on the 6-week CDAHFD feeding duration and the histological findings, this model represents early to mild-to-moderate fibrosis rather than mature fibrosis or cirrhosis (17). Sirius Red staining revealed a significant increase in hepatic fibrosis area in MASLD mice. Both ATS and MI treatments markedly reduced fibrosis area (Figures 4A,D). Immunohistochemical staining further demonstrated that the positive areas for COL1A1 and α-SMA were significantly elevated in MASLD mice, which were similarly reduced by ATS and MI treatment (Figures 4B,C, E, F). The effects of ATS and MI showed no significant difference in this model. These results collectively indicate that MI significantly attenuates hepatic fibrosis in MASLD mice, demonstrating an effect comparable to that of ATS in this experimental model.

**Figure 4:**
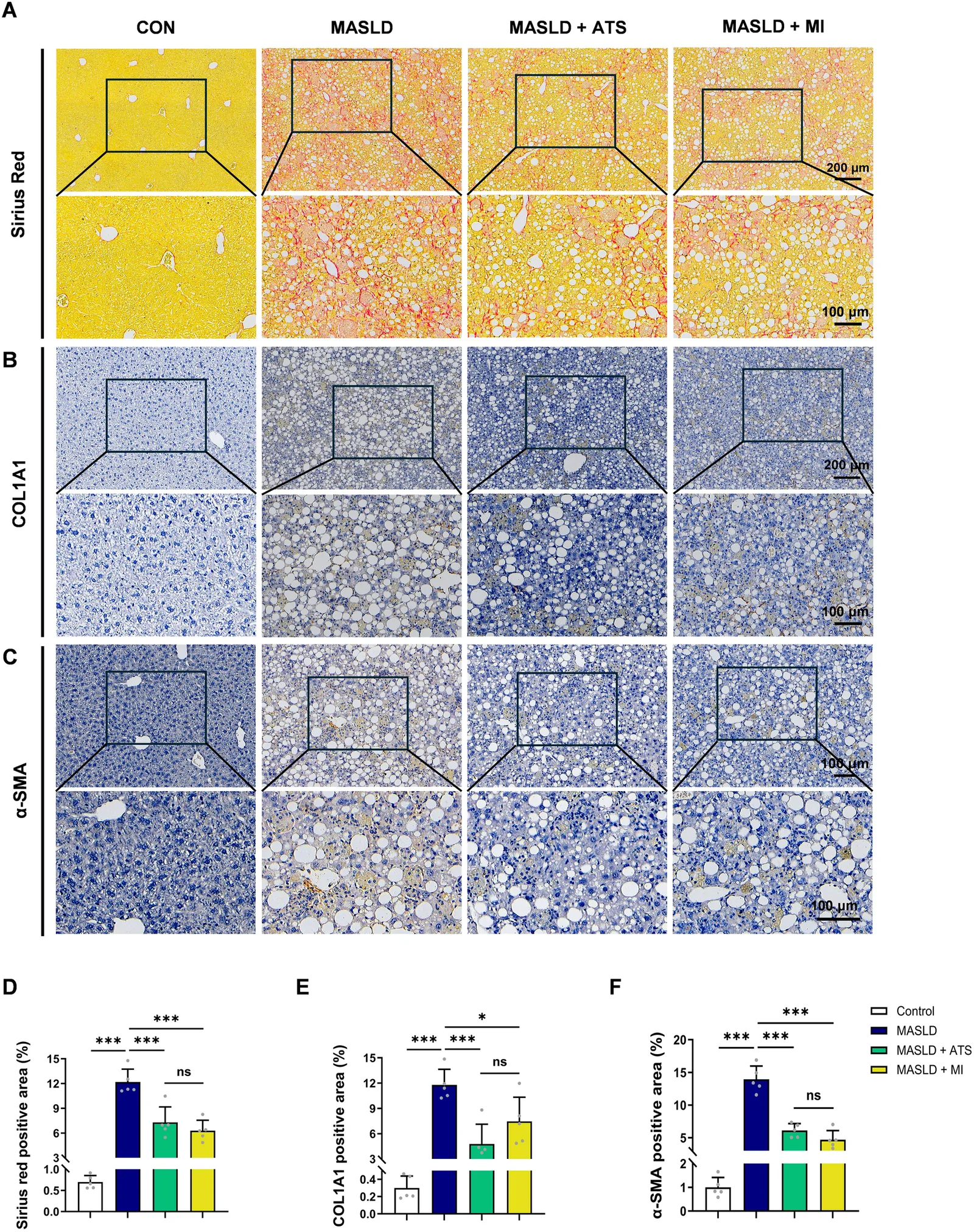
MI treatment (4 mg/kg/day) attenuated hepatic fibrosis in MASLD mice (*n* = 5 biological replicates per group). **(A,D)** Representative images of Sirius Red staining and statistical analysis of hepatic fibrosis area; **(B,C,E,F)** Representative immunohistochemical staining images of COL1A1 and α-SMA and statistical analysis of positive areas. Data are presented as mean ± SD. Statistical significance was determined by one-way ANOVA with Bonferroni's comparison test. Horizontal lines represent the statistical comparisons between the two connected groups. ns, not significant, *P < 0.05, ***P < 0.001.

### 3.4 MI treatment mitigated oxidative stress

Compared with the control group, mice in the MASLD group exhibited a significant increase in the mean fluorescence intensity of reactive oxygen species (ROS) in liver tissue. Both ATS and MI interventions markedly attenuated this increase, with no statistically significant difference observed between the two treatments (Figures 5A,B). Assays of MDA and SOD revealed that both ATS and MI effectively lowered hepatic MDA levels and enhanced SOD activity in MASLD mice, with ATS showing a more pronounced effect than MI under the present experimental conditions (Figures 5C,D). SOD can scavenge lipid peroxides and free radicals, thereby reducing lipid peroxidation. MDA, an end product of lipid peroxidation, serves as a well-established biomarker for assessing oxidative damage. The alterations in SOD and MDA levels indicate that MI exerts antioxidative protective effects in the liver of MASLD mice, although its effects were less pronounced compared to ATS in this experimental model.

**Figure 5:**
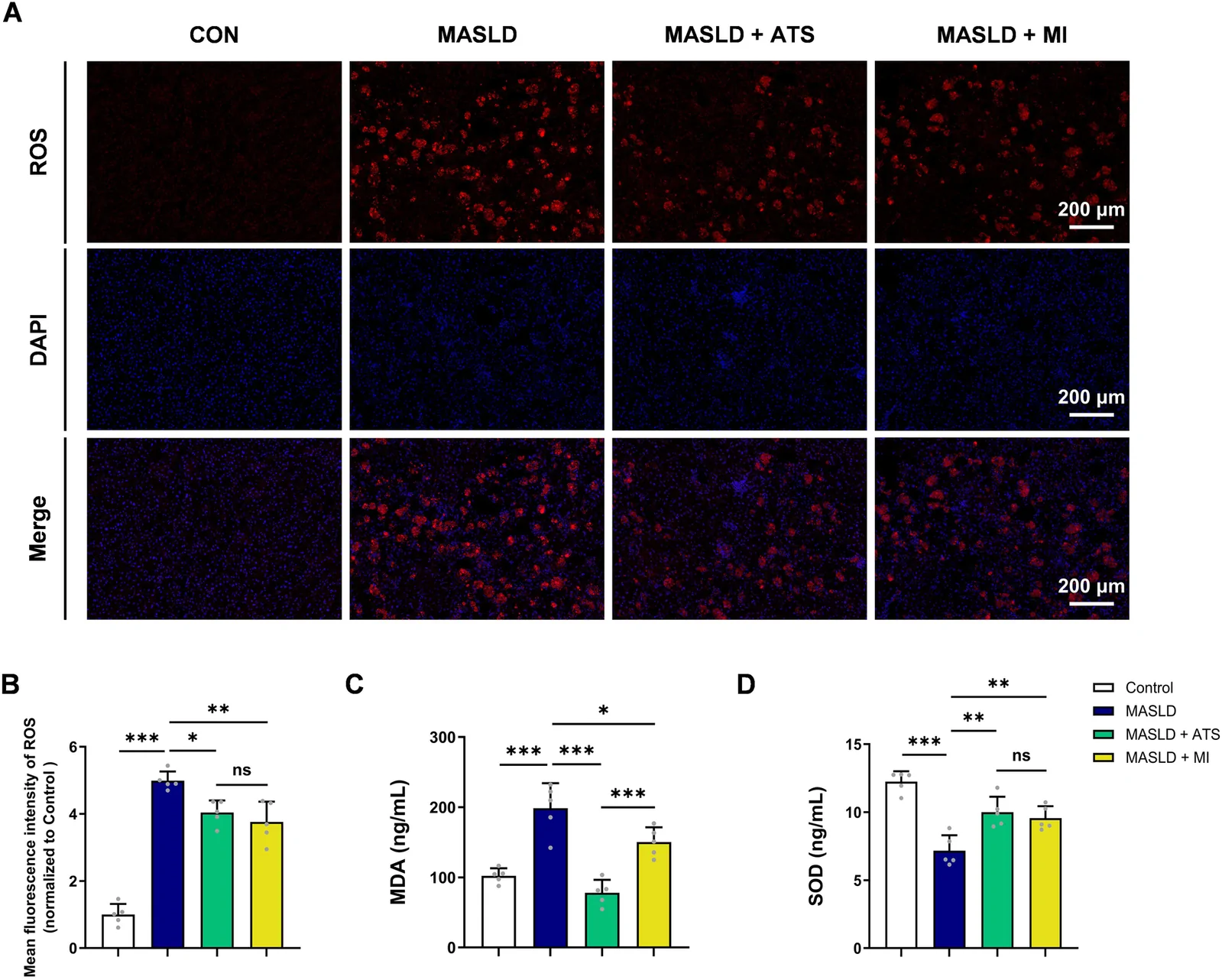
MI treatment (4 mg/kg/day) mitigated oxidative stress in MASLD mice (*n* = 5 biological replicates per group). **(A,B)** O2- generation was detected via dihydroethidium staining and mean fluorescence intensity was calculated; **(C,D)** Levels of hepatic MDA and SOD. Data are presented as mean ± SD. Statistical significance was determined by one-way ANOVA with Bonferroni's comparison test. Horizontal lines represent the statistical comparisons between the two connected groups. ns, not significant, *P < 0.05, **P < 0.01, ***P < 0.001.

### 3.5 MI treatment modulated the cholesterol transcriptome and the AR/SCAP/SREBP2 signaling pathway

Transcriptomic analysis identified 522 differentially expressed genes (DEGs) between the control group and the MASLD group, among which 219 were up-regulated and 303 were down-regulated. Several genes related to lipid biosynthesis were up-regulated in the MASLD group (Figure 6A). Between the MASLD group and the MASLD + MI group, 444 DEGs were detected (214 up-regulated and 230 down-regulated), and some genes associated with cholesterol biosynthesis were down-regulated in the MI group (Figure 6B). GO analysis showed that genes up-regulated in the MASLD group were enriched in biological processes such as acyl-CoA metabolism, fatty acid metabolism, lipid metabolism, and lipid metabolism-related enzyme activities, whereas genes down-regulated after MI treatment were enriched in steroid biosynthetic process, acyl-CoA metabolic process, and lipid metabolic process. KEGG pathway enrichment analysis revealed that up-regulated genes in the MASLD group were enriched in pathways including biosynthesis of unsaturated fatty acids, PPAR signaling pathway, fatty acid elongation, and butanoate metabolism. The top three significantly enriched pathways among genes down-regulated after MI intervention were biosynthesis of unsaturated fatty acids, fatty acid elongation, and PPAR signaling pathway (Figures 6C,D). These data indicate that the conventional modeling approach induces elevated expression of lipid metabolism-related genes, and that MI potentially ameliorates MASLD by influencing steroid metabolism and lipid metabolism. The biosynthesis of steroid and fatty acids is a complex process in which SREBPs (SREBP1/2) and SCAP serve as the core regulators. They sense cellular demands and control the entire biosynthetic process by directing the expression of downstream enzyme genes (18). In particular, SREBP2 primarily activates cholesterol synthesis genes but can also activate genes encoding enzymes required for fatty acid synthesis, such as stearoyl-CoA desaturase (19). Therefore, we examined the AR/SCAP/SREBP2 signaling pathway to further explore the mechanism by which MI affects hepatic cholesterol metabolism.

**Figure 6:**
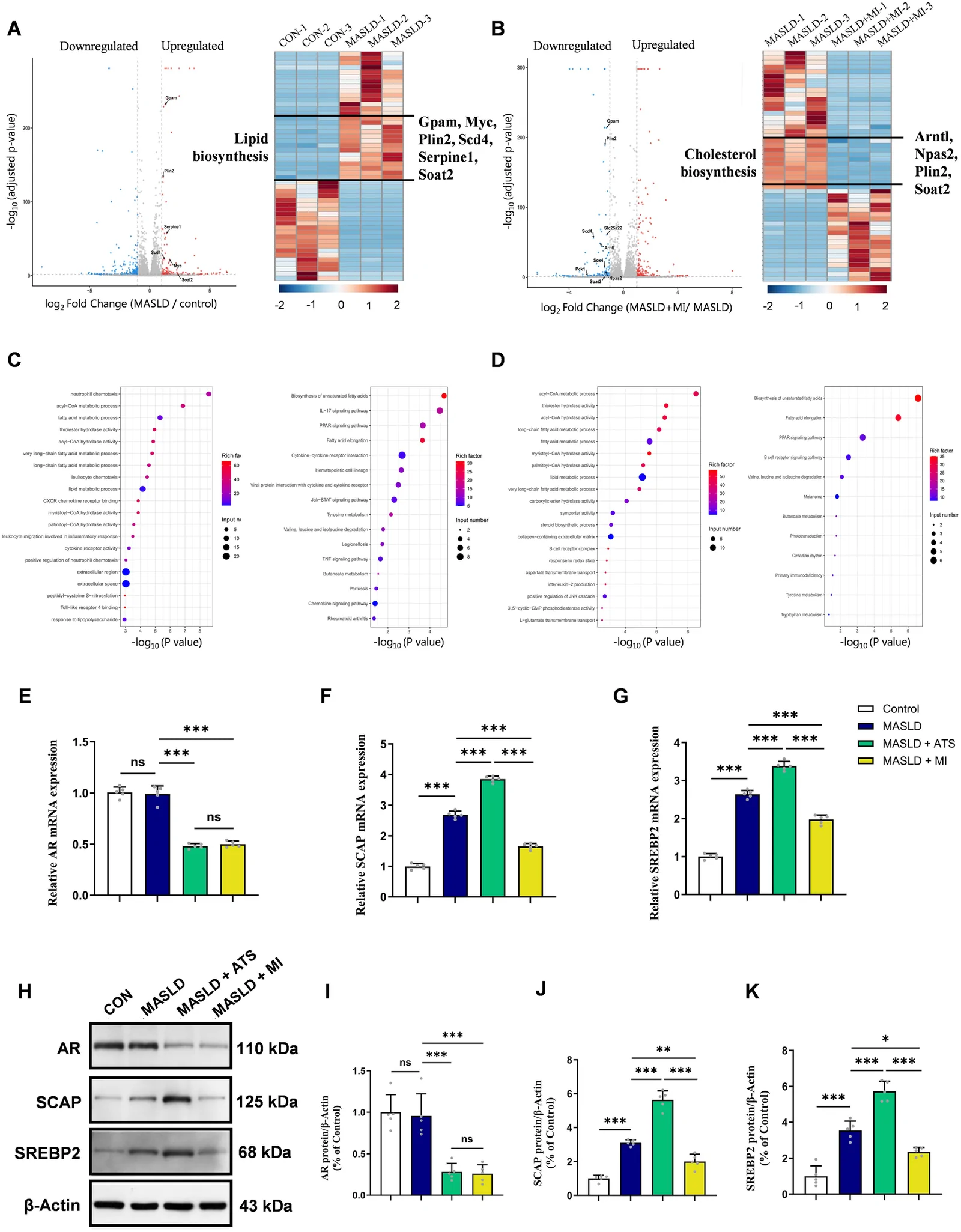
MI treatment (4 mg/kg/day) modulated the cholesterol transcriptome and the AR/SCAP/SREBP2 signaling pathway in the liver of MASLD mice. **(A)** Left: Volcano plot of DEGs in control and MASLD; Right: DEG heatmap (*n* = 3 biological replicates per group); **(B)** Left: Volcano plot of DEGs in MASLD and MASLD + MI; Right: DEG heatmap (*n* = 3 biological replicates per group); **(C)** GO and KEGG analysis between control and MASLD groups (*n* = 3 biological replicates per group); **(D)** GO and KEGG analysis between MASLD and MASLD + MI groups (*n* = 3 biological replicates per group); **(E,F,G)** AR, SCAP and SREBP2 were detected using qRT-PCR (*n* = 5 biological replicates per group); **(H–K)** Results of western blotting of AR, SCAP and SREBP2 in liver (*n* = 5 biological replicates per group). For RNA-seq, three biological replicates per group were randomly selected from five animals per group. For qRT-PCR and Western blot, five biological replicates per group were used; among these, three samples per group overlapped with the RNA-seq samples and two samples per group were not used for RNA-seq. Data are presented as mean ± SD. Statistical significance was determined by one-way ANOVA with Bonferroni's comparison test. Horizontal lines represent the statistical comparisons between the two connected groups. ns, not significant, *P < 0.05, **P < 0.01, ***P < 0.001.

Results from qRT-PCR and Western blot analyses revealed that expression levels of SREBP cleavage-activating protein (SCAP) and sterol regulatory element-binding protein 2 (SREBP2) were significantly elevated in the MASLD group compared with the control group (*p* < 0.001 for all comparisons), while AR levels showed no significant change (Figures 6E–K). In the ATS-treated group, the expression of SCAP and SREBP2 was markedly higher than in both the control and MASLD groups. Conversely, AR levels in the ATS group were significantly downregulated compared to the MASLD group. In contrast, MI intervention significantly downregulated the mRNA and protein expression levels of AR, SCAP, and SREBP2 (PCR: AR, SCAP and SREBP2, all *p* < 0.001; WB: AR, *p* < 0.001; SCAP, *p* = 0.001; SREBP2, *p* = 0.01). These findings suggest that the elevated cholesterol levels observed in MASLD mice may be partly attributed to increased SREBP2 protein, which aligns with previous study. MI treatment was associated with reduced hepatic cholesterol levels and decreased SCAP/SREBP2 expression in MASLD mice. These effects may involve mechanisms distinct from those of ATS. However, causality was not established. Furthermore, although MI reduced AR levels, the absence of AR upregulation in the MASLD group indicates that the cholesterol-lowering effect of MI is not mediated through AR suppression.

## 4 Discussion

In this study, we demonstrated for the first time that MI, an active component of GXD, significantly ameliorated hepatic cholesterol accumulation, inflammation, fibrosis and oxidative stress in a mouse model of MASLD. Beyond systematically characterizing its pleiotropic effects, a major finding of our work lies in the association between these protective actions and reduced expression of the SCAP/SREBP2 cholesterol metabolic signaling pathway, thereby offering a potential perspective for elucidating its underlying mechanism.

Our results showed that MI effectively improved cholesterol metabolic disorders in MASLD mice, as evidenced by significant reductions in liver index, hepatic TC and FC content. However, MI did not improve the reduction in serum HDL-C in model mice. This finding is consistent with previous reports showing that interventions targeting hepatic cholesterol metabolism can reduce liver cholesterol independently of circulating HDL-C (20, 21). This lack of improvement may reflect a balance between reduced hepatic cholesterol availability for HDL assembly and preserved or compensatory cholesterol efflux pathways. The amelioration of cholesterol metabolic disorders is particularly noteworthy given that FC is highly toxic to multiple cellular processes and liver lipid droplet cholesterol content is a key determinant of MASLD (22). This is consistent with previous reports that *Allium* macrostemon and its saponins inhibit lipid peroxidation and ameliorate hyperlipidemia (23, 24). However, earlier mechanistic studies have largely focused on downstream effects, leaving the role of this compound in regulating core transcription factors in cholesterol metabolism unexplored. SREBPs are pivotal hubs controlling lipid synthesis and uptake and their dysregulation occurs in various metabolic syndromes and cancers (25–27). Aberrant sustained activation of the SREBP2 simultaneously enhances the synthesis and transportation of endogenous cholesterol. This activation is a core pathogenic driver of cholesterol overload in MASLD, contributing to inflammation and cell death (28). Our study revealed that MI significantly suppressed SREBP2 protein expression, which partially aligns with the finding that notoginsenoside R1 attenuated hepatic steatosis by inhibiting SREBP2 (29). This suggests that targeting SREBP2 may be a potential strategy for MASLD treatment.

More importantly, a novel aspect of our study is that the inhibitory effect of MI may originate from its upstream regulator SCAP. SCAP is a polytopic membrane protein that mediates the transport and activation of SREBP2 from the endoplasmic reticulum to the Golgi apparatus. It acts as a crucial cholesterol sensor in mammalian cells (30). Although SCAP is essential for cholesterol homeostasis, its direct exploration in MASLD pathogenesis has been limited. Importantly, multiple evidences support the functional significance of SCAP in regulating SREBP2 activation. siRNA-mediated knockdown of SCAP inhibits SREBP2 cleavage, and myeloid-specific SCAP deficiency impedes SREBP2 maturation (31). Moreover, SCAP-KO cells exhibit drastically reduced SREBP2 protein levels and down-regulated SREBP2 target gene expression, while pharmacological SCAP inhibition blocks SREBP2 maturation by promoting SCAP-Insig interaction (32, 33). In the liver, specific deletion of SCAP results in a 90% reduction of fatty acid synthesis rates, highlighting its critical role in hepatic lipid metabolism (27). Thus, we propose that the “SCAP-SREBP2” signaling axis may be a potentially involved pathway in the effects of MI, which may add to existing knowledge. Nevertheless, we acknowledge that reduced SCAP expression alone may not fully reflect SCAP functional activity, and future studies measuring SREBP2 level and SCAP-SREBP2 complex formation will be necessary to definitively establish this mechanism. In addition, an apolipoprotein J (ApoJ) antagonist peptide has been shown to facilitate ubiquitin-proteasomal degradation of mammalian target of rapamycin (mTOR), restore lipophagy and lysosomal activity, and thereby prevent hepatic lipid deposition and improve metabolic homeostasis in MASLD mice (34). However, whether MI converges with the ApoJ–mTOR pathway requires further investigation.

Having established its regulation at the metabolic source, our findings strongly support the pathological model of “lipotoxicity-driven inflammation and fibrosis.” In this study, while inhibiting the SCAP/SREBP2 pathway, MI markedly alleviated liver inflammation, as evidenced by downregulation of pro-inflammatory cytokines IL-1β, IL-6, and TNF-α, and reduced expression of CD11b, a broad marker of myeloid cells, suggesting decreased hepatic myeloid cell infiltration, consistent with attenuated liver inflammation. Activated myeloid cells, such as macrophages and neutrophils, secrete a variety of bioactive mediators that play significant roles in liver injury. These mediators can differentiate quiescent hepatic stellate cells (HSCs) into myofibroblasts that are characterized by their high synthetic output of extracellular matrix (35). It has been widely reported that disruption of cholesterol homeostasis and the accumulation of oxysterols are key drivers of hepatic inflammation (36, 37). Consistent with this concept, adiponectin-derived alkaline phosphatase (ALP)-activated self-assembling peptides that reduce lipid accumulation and hepatic steatosis in high-fat diet (HFD)-induced MASLD also modulate inflammatory pathways according to transcriptomic analysis, supporting that alleviating upstream lipid metabolic dysregulation can indirectly attenuate hepatic inflammation (38). Therefore, the anti-inflammatory effects we observed likely result not from direct inhibition of inflammatory pathways, but as a secondary benefit of reducing upstream lipotoxic substrates.

Similarly, the significant decreases in markers of fibrosis, including α-SMA, COL1A1, and collagen area, are a logical extension of this mechanism. This pattern of modulating the “metabolism–inflammation–fibrosis” cascade at its source may have more fundamental therapeutic significance and translational potential than simply blocking a downstream inflammatory mediator alone. It should be noted that the CDAHFD model used in this study primarily recapitulates early to mild-to-moderate fibrosis rather than established/mature fibrosis or cirrhosis. Therefore, the reduced Sirius Red-positive area and decreased COL1A1/α-SMA staining after 30 days of treatment should be interpreted as attenuation of early fibrotic changes, rather than reversal of advanced fibrosis. Longer-term studies will be needed to determine whether this intervention can reverse mature fibrosis or cirrhosis.

Furthermore, our study demonstrated that MI significantly ameliorated hepatic oxidative stress, as reflected by increased SOD activity and reduced levels of MDA and ROS. Oxidative stress represents another pivotal factor in the progression of MASLD, which is closely linked to lipid peroxidation (39). The present findings align with the established antioxidant capacity of *Allium* macrostemon extracts (40). This antioxidant effect may be mechanistically linked to the antifibrotic phenotype described above, because oxidative stress can induce the redox-sensitive chaperone ApoJ in hepatocytes, and hepatocyte ApoJ has been reported to promote HSC activation and liver fibrosis via the RanBP2/STAT3/TGF-β/ROS axis (41). Therefore, by restoring redox balance, MI may indirectly attenuate this oxidative stress–ApoJ–STAT3–HSC profibrotic cascade, although direct evidence that MI modulates this pathway is still lacking. A novel aspect of our study lies in correlating the observed antioxidant effects with specific metabolic pathways. By inhibiting the SCAP/SREBP2 signaling pathway and its downstream cholesterol synthesis, MI may directly reduce oxidizable lipid substrates. Moreover, modulation of SCAP/SREBP2 activity could influence ROS-producing pathways such as NADPH oxidase (42). Therefore, the antioxidant action of MI may not be an isolated event, but rather part of its integrated metabolic regulatory effects.

Our study has certain limitations. First, the relatively small sample size may limit the statistical power to detect small effect sizes. Second, our evidence for the SCAP/SREBP2 pathway is primarily based on expression changes following pharmacological inhibition and logical inference; more direct genetic validation using liver-specific SREBP2 knockout or overexpression mouse models is warranted in the future. Total SREBP2 expression does not fully reflect pathway activation, as we did not distinguish precursor from mature/nuclear SREBP2 or assess downstream targets such as HMGCR and LDLR. Future studies are needed to validate the causal role of this pathway. Third, the direct binding or inhibitory action of MI on SCAP remains to be confirmed by molecular docking and *in vitro* binding assays. Finally, the *in vivo* pharmacokinetic profile and liver-targeting properties of MI need to be characterized, which is a crucial step toward clinical translation. Further investigations along these directions are underway.

## 5 Conclusions

In summary, this study investigated the effects of MI intervention on disease progression and its underlying mechanisms using a mouse model of MASLD. Our findings demonstrated that MI alleviated hepatic cholesterol accumulation, liver injury, inflammation, oxidative stress, and fibrosis in MASLD mice. To our knowledge, this study is the first to show that its regulatory effect on cholesterol metabolism is associated with reduced expression of the hepatic SCAP/SREBP2 signaling pathway, although its effects on some parameters were less pronounced than those of the positive control ATS in this experimental model. This research provided important preclinical evidence that MI may exert beneficial effects in an animal model of MASLD and suggested that the SCAP/SREBP2 pathway may be a potentially involved pathway.

A limitation of this study is the relatively small sample size that may limit the statistical power. Second, the lack of more direct mechanistic validation, such as through SREBP2 gene knockout or overexpression experiments at cellular and animal levels, which will be the focus of our future research. Meanwhile, total SREBP2 expression does not fully represent pathway activation, and mature/nuclear SREBP2 and downstream cholesterol-biosynthesis targets were not assessed. Therefore, the causal role of SCAP/SREBP2 in mediating the effects of MI remains to be validated. Additionally, further studies are needed to verify its efficacy in clinical samples and to explore the specific downstream effector network of this pathway.

## Data Availability

The datasets presented in this study can be found in online repositories. The names of the repository/repositories and accession number(s) can be found below: https://www.ncbi.nlm.nih.gov/, PRJNA1533871.

## References

[1] Paik JM Henry L Younossi Y Ong J Alqahtani S Younossi ZM. The burden of nonalcoholic fatty liver disease (nafld) is rapidly growing in every region of the world from 1990 to 2019. Hepatol Commun. (2023) 7(10):e0251. 10.1097/hc9.0000000000000251 37782469 PMC10545420

[2] Duell PB Welty FK Miller M Chait A Hammond G Ahmad Z. Nonalcoholic fatty liver disease and cardiovascular risk: a scientific statement from the American Heart Association. Arterioscler Thromb Vasc Biol. (2022) 42(6):e168–e85. 10.1161/atv.0000000000000153 35418240

[3] Pouwels S Sakran N Graham Y Leal A Pintar T Yang W. Non-alcoholic fatty liver disease (nafld): a review of pathophysiology, clinical management and effects of weight loss. BMC Endocr Disord. (2022) 22(1):63. 10.1186/s12902-022-00980-1 35287643 PMC8919523

[4] Eslam M Newsome PN Sarin SK Anstee QM Targher G Romero-Gomez M. A new definition for metabolic dysfunction-associated fatty liver disease: an international expert consensus statement. J Hepatol. (2020) 73(1):202–9. 10.1016/j.jhep.2020.03.039 32278004

[5] Chan W-K Tan S-S Chan S-P Lee Y-Y Tee H-P Mahadeva S. Malaysian society of gastroenterology and hepatology consensus statement on metabolic dysfunction-associated fatty liver disease. J Gastroenterol Hepatol. (2022) 37(5):795–811. 10.1111/jgh.15787 35080048 PMC9303255

[6] Maxfield FR Tabas I. Role of cholesterol and lipid organization in disease. Nature. (2005) 438(7068):612–21. 10.1038/nature04399 16319881

[7] Weidner C Braun LT. Risk factors for atherosclerotic cardiovascular disease. In: Dunbar SB Braun LT, editors. Preventive Cardiovascular Nursing: Resilience Across the Lifespan for Optimal Cardiovascular Wellness. Cham: Springer International Publishing (2024). p. 85–120.

[8] McGettigan B McMahan R Orlicky D Burchill M Danhorn T Francis P. Dietary lipids differentially shape nonalcoholic steatohepatitis progression and the transcriptome of Kupffer cells and infiltrating macrophages. Hepatology. (2019) 70(1):67–83. 10.1002/hep.30401 30516830 PMC6923128

[9] Zhou H Ma C Wang C Gong L Zhang Y Li Y. Research progress in use of traditional Chinese medicine monomer for treatment of non-alcoholic fatty liver disease. Eur J Pharmacol. (2021) 898:173976. 10.1016/j.ejphar.2021.173976 33639194

[10] Yao ZH Qin ZF Dai Y Yao XS. Phytochemistry and pharmacology of allii macrostemonis bulbus, a traditional Chinese medicine. Chin J Nat Med. (2016) 14(7):481–98. 10.1016/s1875-5364(16)30058-9 27507199

[11] Guo JE Mi SB Yan XC Xin SY Gao F Liang GH. Effects of Gualou Xiebai Banxia decoction on blood lipid content, oxidative stress and Ox-Ldl/Lox-1 pathway in apoe-/- mice. Zhongguo Zhong Yao Za Zhi. (2017) 42(4):752–7. 10.19540/j.cnki.cjcmm.20161222.076 28959848

[12] Li QH Su JY Ding R He ZQ Liang C Wu ZG. Effects of tanyu tongzhi prescription on lipid metabolism in hyperlipidemic mice and its mechanisms. Chin J Integrat Med Cardio-Cerebrovasc Dis. (2017) 15(6):663–6. (in Chinese).

[13] Wang H Zheng Q Dong A Wang J Si J. Chemical constituents, biological activities, and proposed biosynthetic pathways of steroidal saponins from healthy nutritious vegetable-allium. Nutrients. (2023) 15(9):2233. 10.3390/nu15092233 37432450 PMC10180908

[14] Xie W Zhang Y Wang N Zhou H Du L Ma X. Novel effects of macrostemonoside a, a compound from allium macrostemon bung, on hyperglycemia, hyperlipidemia, and visceral obesity in high-fat diet-fed C57bl/6 mice. Eur J Pharmacol. (2008) 599(1–3):159–65. 10.1016/j.ejphar.2008.09.042 18930725

[15] Peng J Yao X Kobayashi H Ma C. Novel furostanol glycosides from allium macrostemon. Planta Med. (1995) 61(1):58–61. 10.1055/s-2006-958000 7700993

[16] Akuta N Kogiso T Ikejima K Otsuka M Kawaguchi T Kawanaka M. Evidence-based clinical practice guidelines for metabolic dysfunction–associated steatotic liver disease (Masld) 2026. Hepatol Res. (2026) 56(6):887–902. 10.1111/hepr.70188 42118685

[17] Hartimath SV Boominathan R Soh V Cheng P Deng X Chong YC. Imaging fibrogenesis in a diet-induced model of nonalcoholic steatohepatitis (Nash). Contrast Media Mol Imaging. (2019) 2019:1–8. 10.1155/2019/6298128 PMC6914933 31866798

[18] Shimano H. Sterol regulatory element-binding proteins (srebps): transcriptional regulators of lipid synthetic genes. Prog Lipid Res. (2001) 40(6):439–52. 10.1016/S0163-7827(01)00010-8 11591434

[19] Pai JT Guryev O Brown MS Goldstein JL. Differential stimulation of cholesterol and unsaturated fatty acid biosynthesis in cells expressing individual nuclear sterol regulatory element-binding proteins. J Biol Chem. (1998) 273(40):26138–48. 10.1074/jbc.273.40.26138 9748295

[20] Liu N Tian J Steer CJ Han Q Song G. Microrna-206-3p suppresses hepatic lipogenesis and cholesterol synthesis while driving cholesterol efflux. Hepatology. (2025) 81(1):111–25. 10.1097/HEP.0000000000000672 37943861

[21] Huang L Wang S Zhang H Feng S Zhong H Chen J. Clinical efficacy evaluation of washed microbiota transplantation treatment for metabolic related fatty liver disease and its impact on tongue coating microorganisms. Front Endocrinol (Lausanne). (2025) 16:2025. 10.3389/fendo.2025.1684173 PMC12605323 41234231

[22] Sakuma I Gaspar RC Nasiri AR Dufour S Kahn M Zheng J. Liver Lipid Droplet Cholesterol Content Is a Key Determinant of Metabolic Dysfunction–Associated Steatohepatitis. Proc. Natl. Acad. Sci. USA. (2025) 122(18):e2502978122. 10.1073/pnas.2502978122 PMC12067271 40310463

[23] Liu L Qiu L Xue J Zhong C Qin M Zhang Y. Saponins from allium macrostemon bulbs attenuate endothelial inflammation and acute lung injury via the Nf-Κb/Vcam-1 pathway. Molecules. (2024) 29(6):1239. 10.3390/molecules29061239 38542876 PMC10976117

[24] Ding W Sun Y Wu J Li H Wang Z Li W. Spirostanol-type saponins from allium macrostemonis bulbus alleviate chronic unpredictable mild stress–induced depression-like behavior in mice. J Food Biochem. (2025) 2025(1):8113079. 10.1155/jfbc/8113079

[25] Wang M Li Y Li S Wang T Wang M Wu H. Cinobufacini injection delays hepatocellular carcinoma progression by regulating lipid metabolism via Srebp1 signaling pathway and affecting macrophage polarization. J Ethnopharmacol. (2024) 321:117472. 10.1016/j.jep.2023.117472 37995825

[26] Jiang G Li J Niu S Dong R Chen Y Bi W. Ly86 facilitates Ox-Ldl-induced lipid accumulation in macrophages by upregulating Srebp2/Hmgcr expression. BMC Cardiovasc Disord. (2024) 24(1):289. 10.1186/s12872-024-03957-1 38822281 PMC11140969

[27] Chandrasekaran P Weiskirchen R. The role of scap/srebp as central regulators of lipid metabolism in hepatic steatosis. Int J Mol Sci. (2024) 25(2):1109. 10.3390/ijms25021109 38256181 PMC10815951

[28] Fan Y-M Zhang Q-Q Pan M Hou Z-F Fu L Xu X. Toxoplasma gondii sustains survival by regulating cholesterol biosynthesis and uptake via Srebp2 activation. J Lipid Res. (2024) 65(12):100684. 10.1016/j.jlr.2024.100684 39490926 PMC11626538

[29] Chen Z Li C Yang C Zhao R Mao X Yu J. Lipid regulation effects of raw and processed notoginseng radix et rhizome on steatotic hepatocyte L02 cell. Biomed Res Int. (2016) 2016:2919034–9. 10.1155/2016/2919034 27642594 PMC5013208

[30] Fan Y Zhang R Wang C Pan M Geng F Zhong Y. Stat3 activation of Scap-Srebp-1 signaling upregulates fatty acid synthesis to promote tumor growth. J Biol Chem. (2024) 300(6):107351. 10.1016/j.jbc.2024.107351 38718868 PMC11176798

[31] Wu Y Zheng K Dong LL Gao S Wu Y Liu Z. Srebp2 promotes macrophage alternative activation and allergic airway inflammation independent of cholesterol biosynthesis. Cell Death Dis. (2026) 17(1):697. 10.1038/s41419-026-08910-y 42236481 PMC13448065

[32] Tang J-J Li J-G Qi W Qiu W-W Li P-S Li B-L. Inhibition of srebp by a small molecule, betulin, improves hyperlipidemia and insulin resistance and reduces atherosclerotic plaques. Cell Metab. (2011) 13(1):44–56. 10.1016/j.cmet.2010.12.004 21195348

[33] Kuan Y-C Takahashi Y Maruyama T Shimizu M Yamauchi Y Sato R. Ring finger protein 5 activates sterol regulatory element–binding protein 2 (Srebp2) to promote cholesterol biosynthesis via inducing polyubiquitination of srebp chaperone scap. J Biol Chem. (2020) 295(12):3918–28. 10.1074/jbc.RA119.011849 32054686 PMC7086040

[34] Duan S Qin N Pi J Sun P Gao Y Liu L. Antagonizing apolipoprotein J chaperone promotes proteasomal degradation of mtor and relieves hepatic lipid deposition. Hepatology. (2023) 78(4):1182–99. 10.1097/HEP.0000000000000185 37013405

[35] Widowati W Sabrina AHN Sutendi AF Zahiroh FH Nurjamil AM Wargasetia TL. Immune system and hepatic stellate cells’ crosstalk in liver fibrosis: pathways and therapeutic potential. J Immunol Res. (2026) 2026:2656395. 10.1155/jimr/2656395 41487942 PMC12759114

[36] Wei X Yin F Wu M Xie Q Zhao X Zhu C. G protein-coupled receptor 35 attenuates nonalcoholic steatohepatitis by reprogramming cholesterol homeostasis in hepatocytes. Acta Pharm Sin B. (2023) 13(3):1128–44. 10.1016/j.apsb.2022.10.011 36970193 PMC10031266

[37] Horn CL Morales AL Savard C Farrell GC Ioannou GN. Role of cholesterol-associated steatohepatitis in the development of Nash. Hepatol Commun. (2022) 6(1):12–35. 10.1002/hep4.1801 34558856 PMC8710790

[38] Li Z Duan S Zhu Z Han H Qin N Ji Q. Enzyme-activated self-assembling peptides mimicking adiponectin multimers for nonalcoholic fatty liver disease therapy. ACS Cent Sci. (2026) 12(2):243–53. 10.1021/acscentsci.5c02405 41768763 PMC12947284

[39] Song S Zheng J Zhao D Zheng A Zhu Y Xu Q. Quantitative proteomics analysis based on data-independent acquisition reveals the effect of shenling baizhu powder (Slp) on protein expression in mafld rat liver tissue. Clin Proteomics. (2023) 20(1):55. 10.1186/s12014-023-09442-9 38036981 PMC10691125

[40] Barbu IA Toma VA Moț AC Vlase AM Butiuc-Keul A Pârvu M. Chemical composition and antioxidant activity of six allium extracts using protein-based biomimetic methods. Antioxidants (Basel). (2024) 13(10):1182. 10.3390/antiox13101182 39456436 PMC11504208

[41] Liu L Mo Z Qin N Pi J Mao L Deng G. Hepatocyte apolipoprotein J accelerates injury-induced liver fibrosis by activation signal transducer and activator of transcription 3 through Ranbp2 mediated-sumoylation. Cell Mol Gastroenterol Hepatol. (2025) 19(10):101556. 10.1016/j.jcmgh.2025.101556 40545037 PMC12391277

[42] Abe J Berk BC. Atheroprone flow activation of the sterol regulatory element binding protein 2 and nod-like receptor protein 3 inflammasome mediates focal atherosclerosis. Circulation. (2013) 128(6):579–82. 10.1161/circulationaha.113.004390 23838164 PMC4091715

