# Supplementary material for: Macrostemonoside I alleviates metabolic dysfunction-associated steatotic liver disease in mice: potential involvement of SCAP/SREBP2 signaling in cholesterol metabolism
Source: Front Med (Lausanne). 2026 Oct 14;13:1983132. doi: 10.3389/fmed.2026.1983132 (PMC13713428; doi:10.3389/fmed.2026.1983132)
Supplement: Supplementary file 1 [file Datasheet1.pdf]

Macrostemonoside I Alleviates Metabolic Dysfunction-Associated Steatotic Liver Disease in Mice: Potential Involvement of SCAP/SREBP2 Signaling in Cholesterol Metabolism

Text S1. Reagents

Atorvastatin (NMPA Approval No. H20051408) was purchased from Pfizer (New York, USA). Mcrostemonoside I (MI) was purchased from MedChemExpress (Princeton, New Jersey, USA, Cat. No. HY-N12148, purity ≥ 98% by HPLC). The powder was stored at -20°C, protected from light and moisture, following the manufacturer's instructions. Rabbit anti-mouse androgen receptor (AR; Cat. No. A19611), rabbit anti-mouse sterol regulatory element-binding protein 2 (SREBP2; Cat. No. A13049), rabbit anti-mouse alpha-smooth muscle actin (α-SMA; Cat. No. A17910), rabbit anti-mouse Collagen I alpha 1 (COL1A1; Cat. No. A24112PM), rabbit anti-mouse CD11b (Cat. No. A26424PM), and rabbit anti-rat β-Actin (Cat. No. AC038) antibodies were purchased from ABclonal Technology (Woburn, MA, USA). Rabbit anti-mouse SREBP cleavage-activating protein (SCAP; Cat. No. GTX639684) was purchased from GeneTex (Irvine, CA, USA). Horseradish peroxidase (HRP)-linked secondary antibody (Cat. No. 7074P2) was purchased from Cell Signaling Technology (Danvers, MA, USA). Commercial assay kits for total cholesterol (TC; Cat. No. CEB701Ge), free cholesterol (FC; Cat. No. EBCK004M), malondialdehyde (MDA; Cat. No. CEA597Ge) and superoxide dismutase (SOD; Cat. No. SEB960Mu) were purchased from Cloud-Clone Corp. (Wuhan, Hubei, China). Enzyme-linked immunosorbent assay (ELISA) kits for tumor necrosis factor alpha (TNF-α; Cat. No. EK282), interleukin-1β (IL-1β; Cat. No. EK201B) and interleukin-6 (IL-6; Cat. No. EK206) were purchased from Multi Sciences (Hangzhou, Zhejiang, China).

Text S2. Detailed protocols for assays

S2.1. Haematoxylin and eosin staining

Liver sections were dewaxed and rehydrated, stained with haematoxylin for 5 min, and rinsed with distilled water. Subsequently, the sections were dehydrated with graded alcohol concentrations (85% and 90%), stained with eosin for 5 min, and cleared in xylene. Sections were first examined under a Nikon microscope (Tokyo, Japan) and then scanned using a Pannoramic MIDI slide scanner (3D Histech, Budapest, Hungary). Five non-overlapping fields per section (central, upper, lower, left, and right) were captured at 10× magnification and then used for qualitative assessment of ballooning degeneration area using Image J 1.8. Fields containing large vessels, bile ducts, capsular edges, tissue folds, or staining artifacts were avoided. The mean of the five fields was used as the final value for each section. Analyses were performed by an investigator blinded to group allocation. Representative image was selected from the section whose final quantitative value was closest to the group mean.

S2.2. Oil Red O Staining

Liver frozen sections were brought to room temperature and fixed in 50% ethanol for 30 min, followed by rinsing with PBS. The sections were then immersed in Oil Red

O working solution for 8 min and differentiated in 50% ethanol for 1 min. Subsequently, the sections were counterstained with hematoxylin for 2 min and mounted with glycerol gelatin mounting medium. The number of fields analyzed per animal, image-selection criteria, blinding, and image analysis software were the same as those described for H&E staining, except that fields were acquired at 20× original magnification and the Oil Red O-positive area was quantified using Image J 1.8. Five non-overlapping fields per animal were analyzed, and the mean of the five fields was used as the final value for each section.

S2.3. Sirius Red staining

The paraffin-embedded sections were dewaxed and stained with haematoxylin for 5 min. The sections were then treated with hydrochloric acid alcohol differentiation solution and rinsed thrice with distilled water. The sections were stained with Sirius red (Sinopharm Chemical Reagent Co., Shanghai, China) for 15 min, dehydrated with absolute ethanol, and sealed with neutral gum. The field number, image-selection criteria, blinding, and image analysis software were consistent with those used for H&E staining. Fields were acquired at 10× original magnification. In each field, the Sirius Red-positive area was quantified using Image J 1.8.

S2.4. Dihydroethidium staining

Oxide (O_2^-) generation was detected using fresh-frozen liver sections incubated with dihydroethidium (Sigma-Aldrich) for 30 min in the dark at room temperature (RT) after quenching spontaneous fluorescence. Sections were first examined under a fluorescence microscope (Nikon) to confirm dihydroethidium fluorescence. Images were then acquired using a Pannoramic MIDI slide scanner. For each section, five images were captured at 10× magnification from the central, upper, lower, left, and right regions. The mean fluorescence intensity of each image was measured using Image J 1.8. The mean fluorescence intensity per section was then calculated as the average of the five image values. The control group mean was used as the normalization reference, and the relative fluorescence intensity of each section was normalized to this control mean.

S2.5. Measurement of hepatic oxidative stress

The supernatant from a 10% homogenate of hepatic tissue was used to measure the MDA and SOD levels following the instructions of commercial kits. The absorbance indices of each group were recorded using a fluorescence microplate reader (Thermo Fisher Scientific, Waltham, MA, USA).

S2.6. Immunohistochemistry

Paraffin-embedded sections were soaked in 3% perhydrol for 25 min in the dark at RT to block endogenous peroxidase activity. Primary antibodies, α-SMA and COL1A1 were incubated overnight at 4℃ after serum blocking with 3% bovine serum albumin (Thermo Fisher Scientific). HRP-linked goat anti-rabbit IgG was incubated for 50 min at RT. Sections were first examined under a Nikon microscope and then scanned using a Pannoramic MIDI slide scanner. The field number, image-selection criteria, blinding,

and image analysis software were consistent with those used for H&E staining. Fields were acquired at 10× original magnification. The COL1A1-positive area and α-SMA-positive area were quantified and expressed as a percentage of the total tissue area. The mean value of the five fields was used as the final quantitative value for each section. Group means were calculated from these section-level values.

S2.7. Immunofluorescent staining

Following deparaffinization and rehydration through xylene and graded ethanol concentrations, the sections underwent antigen retrieval in sodium citrate buffer and were subsequently blocked with bovine serum albumin (BSA). Then sections were incubated with anti-CD11b primary antibody overnight at 4°C, followed by Alexa Fluor 594-conjugated goat anti-rabbit secondary antibody at room temperature for 1 hour in the dark. Nuclei were counterstained with Hoechst. A Nikon fluorescence microscope was used to obtain the images, which were analysed using Image J 1.8.

S2.8. RNA-seq data generation and analysis

Three biological replicates per group were randomly selected from five animals per group. Total RNA was extracted from each sample using TRIzol reagent. RNA concentration and integrity were assessed with a NanoDrop spectrophotometer and an Agilent 2100 Bioanalyzer. Only RNA samples with OD260/280 of 1.8-2.0, OD260/230 > 1.8, and RNA integrity number ≥ 7.0 were used for library construction.

For each sample, 1 μg total RNA was used for mRNA enrichment and library preparation with the Universal V10 RNA-seq Library Prep Kit. Libraries were pooled according to the targeted data output and sequenced on the DNBSEQ-T7 platform (MGI Tech, Shenzhen, China). Unique molecular identifiers (UMIs) were incorporated during library preparation. The sequencing data per sample ranged from 7.3 to 9.75 Gb.

Raw sequencing data were first filtered with fastp (v0.23.2). Low-quality reads were discarded, and adapter sequences were trimmed. Clean reads were further processed with in-house scripts to eliminate duplication bias introduced during library preparation and sequencing. Briefly, clean reads were clustered according to UMI sequences, and reads with the same UMI were grouped into the same cluster. Reads within the same cluster were compared by pairwise alignment, and reads with sequence identity >95% were extracted into a new sub-cluster. After all sub-clusters were generated, multiple sequence alignment was performed to obtain one consensus sequence for each sub-cluster. This step removed errors and biases introduced by PCR amplification or sequencing. The resulting consensus reads were used for standard mRNA-seq analysis.

Reads were aligned to the mouse mm10 reference genome using STAR (v2.7.6a). Reads mapped to the exon regions of each gene and reads mapped to each trascript were counted by featureCounts respectively (Subread-1.5.1; Bioconductor) and then expression was calculated.

Differentially expressed genes (DEGs) and differentially expressed transcripts between groups were identified using edgeR (v3.40.2) with |log2FC| ≥ 1 and Benjamini–Hochberg adjusted P [FDR < 0.05].

Gene ontology (GO) analysis and Kyoto encyclopedia of genes and genomes (KEGG)

enrichment analysis for differentially expressed genes were both implemented by KOBAS software (version: 2.1.1) with a P value cutoff of 0.05. Gene Set Enrichment Analysis (GSEA) was performed using GSEA software with GO and KEGG background datasets from the MSigDB database. Protein－protein interaction networks of DEGs were analyzed using the STRING database. Transcription factors were annotated AnimalTFDB. Individual SNPs and InDels were detected using bcftools (v1.17) and filtered with the bcftools filter module. Filtered SNPs and InDels were annotated using the Variant Effect Predictor (VEP, v106.1). Alternative splicing events were detected using rMATS (v4.1.2) with a P value cutoff of 0.05 and an absolute $\Delta\psi$ value of 0.1. After alignment, reads were assembled de novo using StringTie (v2.1.4). New transcripts were obtained by discarding reads with class code ‘=’. Time-series transcriptome data were clustered using the R package Mfuzz (v2.58.0) to delineate groups of genes with similar expression patterns.

S2.9. Quantitative real-time polymerase chain reaction

All five biological replicates per group were used. Among these, three samples per group overlapped with the RNA-seq samples, and two samples per group were not subjected to RNA-seq. TRIzol reagent (Takara, Osaka, Japan) was used to purify the total RNA from liver tissues and was subsequently reverse transcribed into cDNA using PrimeScript RT Master Mix (Takara) according to the manufacturer’s instructions. On an ABI QuantStudio 3 System (Thermo Fisher Scientific), quantitative real-time polymerase chain reaction (qRT-PCR) was performed using EnTurbo™ SYBR Green PCR SuperMix (Cat. No. EQ001, ELK Biotechnology, Wuhan, Hubei, China). Each reaction was performed in a total volume of 10 μL, containing 1 μL cDNA, 5 μL 2×master mix, 1 μL each of forward and reverse primers (2.5 μM), 2 μL ddH$_2$O, and 1 μL Rox. The amplification conditions were as follows: initial denaturation at 95 °C for 3 min; 40 cycles of 95 °C for 10 s, 58 °C for 30 s, and 72 °C for 30 s; followed by melt curve analysis. All samples were run in triplicate. All primer sequences are listed in Table S1. Relative expression levels of each gene were normalised to β-Actin with $2^{-\Delta\Delta CT}$ method: ΔCT = CT (target) − CT (β-Actin); $\Delta\Delta CT$ = ΔCT (sample) − ΔCT (control); relative expression = $2^{-\Delta\Delta CT}$.

S2.10. Western blotting

All five biological replicates per group were used. Hepatic tissue fragments were lysed with RIPA buffer, and the Bradford protein assay kit (Takara) was used to determine the total protein concentrations as per the manufacturer’s instructions. Samples were loaded at 5–10 μL per lane, with loading volumes adjusted based on preliminary β-actin results to ensure comparable loading, and separated by a 10% Tris-glycine gradient gel and then transferred to polyvinylidene fluoride membranes. Thereafter, the membranes were blocked in PBS containing 5% non-fat dried milk for 2 h at RT. Membranes were incubated with primary antibodies, AR (1:2000), SCAP (1:3000), SREBP2 (1:1000), and β-actin (1:10000), overnight at 4°C and then incubated with HRP-linked IgGs for 2 h at RT after washing with TBST. Chemiluminescence signal was detected using Odyssey Fc Imaging System by Image Studio software (LI-

COR Biosciences, Lincoln, NE, USA). Band intensities were quantified using Image J 1.8. The ratio of target protein to β-actin was calculated for each sample; the mean ratio in the control group was set as the reference, and the ratios of the other groups were normalized to this control mean.

Table S1. The sequence of primer used for reverse transcription.

Gene	Forward	Reverse
AR	5'- GACTCTGGGAGCTCGTAAGC -3'	5'- ACACCACTCCTGGCTCAATG -3'
SCAP	5'- GACTGGATGACTCGTGTTGCC -3'	5'- GATGAAGTCATCCAGGCCACTA -3'
SREBP2	5'- TGACGCAAAGGTCAAGGATG -3'	5'- AAGGAAGTCAAGGGGTTAAAGG -3'
β-Actin	5'- CTGAGAGGGAAATCGTGCGT -3'	5'- CCACAGGATTCCATACCCAAGA -3'
